# Internal Hernia Post-Single Anastomosis Gastric Bypass: Case Series with Review of Literature

**DOI:** 10.1055/s-0044-1788065

**Published:** 2024-07-04

**Authors:** Abdulmenem Abualsel, Raja Nadeem, Fatema Abdulkarim AL-Ahmed, Ebrahim Adel Almahmeed, Roshan George Varkey, Sameer Almobarak, Ajaz A. Wani

**Affiliations:** 1 Divison of General and Bariatric Surgery, Department of Surgical Specialties, King Hamad University Hospital, Al Sayh, Bahrain

**Keywords:** single anastomosis gastric bypass, contrast-enhanced computerized tomography, Roux-en-Y gastric bypass, excess weight loss, internal hernia

## Abstract

Obesity is an emerging worldwide health care issue. It has a direct and indirect bearing on health-related outcomes. Rates of overweight and obesity have grown manifold in the past few decades globally. Once considered a problem of the affluent societies only, obesity is now dramatically on the rise in low- and middle-income countries also. Single anastomosis gastric bypass (SAGB) is one of the combined bariatric procedures adopted for weight loss in patients failing maximal medical therapy. Internal hernia (IH) after SAGB is a less recognized clinical entity. We hereby report our experience with four such cases under light of current available literature. Bariatric procedures are associated with some short- and long-term limitations. IHs are among one of the dreaded complications associated with some bariatric procedures with rates reaching up to 16% after classic Roux-en-Y gastric bypass. The incidence of IH post-SAGB is comparatively rare and is very less frequently reported. Symptoms of IH post-SAGB are quite nonspecific and depend on the time and extent of herniation. The symptoms can vary from benign intermittent colicky pain to severe intra-abdominal pain presenting as a surgical emergency. Routine physical examination and biochemical investigations are nonspecific and unreliable in evaluating those patients. Computed tomography (CT) with intravenous and oral contrast is the most common imaging modality used for preoperative evaluation of those symptoms. The CT findings can be unremarkable in patients having intermittent symptoms/herniation. Diagnostic laparoscopy is the cornerstone for diagnosis and management of patients having high suspicion of IH.

Obesity as defined by the World Health Organization is an abnormal or excessive fat accumulation that imparts direct and indirect health-related risk. It is an emerging concern worldwide including the Middle Eastern countries. Obesity rates in Bahrain are higher in females (38%) than males (20%). 1

The single anastomosis gastric bypass (SAGB) is one among the combined bariatric procedures adopted for weight loss in patients failing maximal medical therapy. It has a good safety profile and is becoming increasingly popular. Internal hernia (IH) refers to the protrusion of bowel through mesenteric defects or spaces. IH most commonly involves the intestinal bowel loops and cannot be visualized externally. High clinical suspicion with symptoms of severe colicky abdominal pain and tachycardia in a patient with known history of gastric bypass should raise suspicion of IH. Classic (Roux-en-Y gastric bypass [RYGB]) gastric bypass is associated with a high incidence of IHs. The risk for IH after classic gastric bypass can be anywhere between 5 and 25%. IH incidence after SAGB is less frequently reported in the literature.

At King Hamad University Hospital, Al Sayh, Bahrain, we adopted the SAGB procedure from early 2013. Between March 2013 and September 2023, 450 patients underwent SAGB with a biliary limb of 150 to 200 cm. We do not perform routine closure of the Petersen's defect after SAGB. Data of patients admitted with clinical suspicion of IH between March 2013 and September 2023 was collected and analyzed. Relevant biochemical and radiological investigations were reviewed.

We hereby present our experience with IHs post-SAGB from a high-volume bariatric surgery center.

## Case Details

### Case 1

A 20-year-old female patient, post-laparoscopic SAGB about 2 years ago presented with severe, periumbilical colicky pain of few days duration. She denied any history of reflux and vomiting. Upon examination she had stable vitals, abdomen was soft and lax, and mild periumbilical and epigastric tenderness with no peritoneal signs.

Routine labs were within normal range. X-ray abdomen was grossly normal. Her previous upper gastrointestinal (GI) endoscopy done few months ago showed healing marginal ulcerations.

Computed tomography (CT) abdomen with contrast was done which reported small bowel jejunojejunal intussusception. There was no evidence of bowel dilatation or obstruction.

She underwent a diagnostic laparoscopy and the intraoperative findings revealed a hyperperistaltic omega loop. The proximal alimentary limb was also distended and the distal small bowel was collapsed. Rotation of the distal alimentary bowel around the mesentery with internal herniation through the Petersen's defect was noted. She underwent reduction of IH and closure of Petersen's defect. Postoperative period was uneventful and she was discharged after 2 days.

**Fig. 1:**
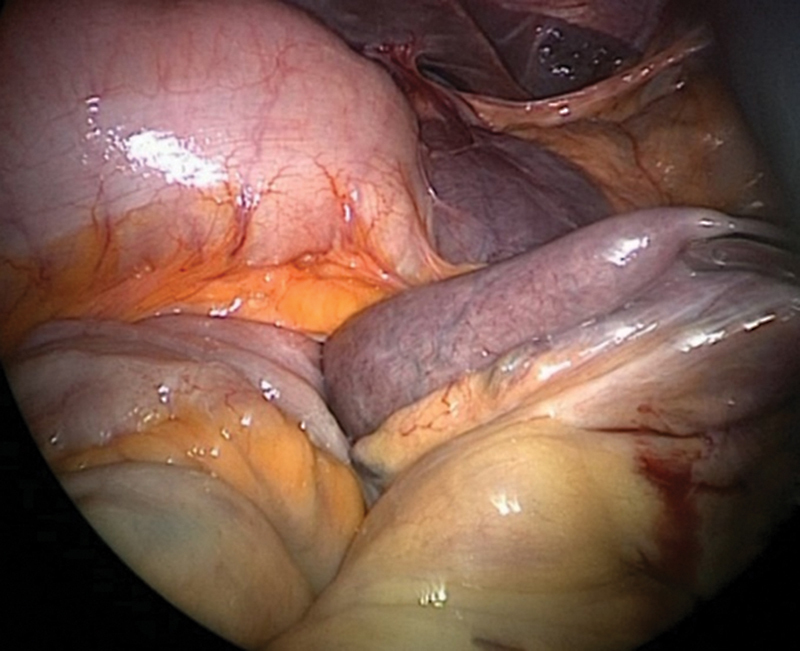
Intraoperative picture of an internal hernia through the Petersen's defect (case 1).

Intraoperative pictures and CT images shown in Figs. 1 2 3.

**Fig. 2:**
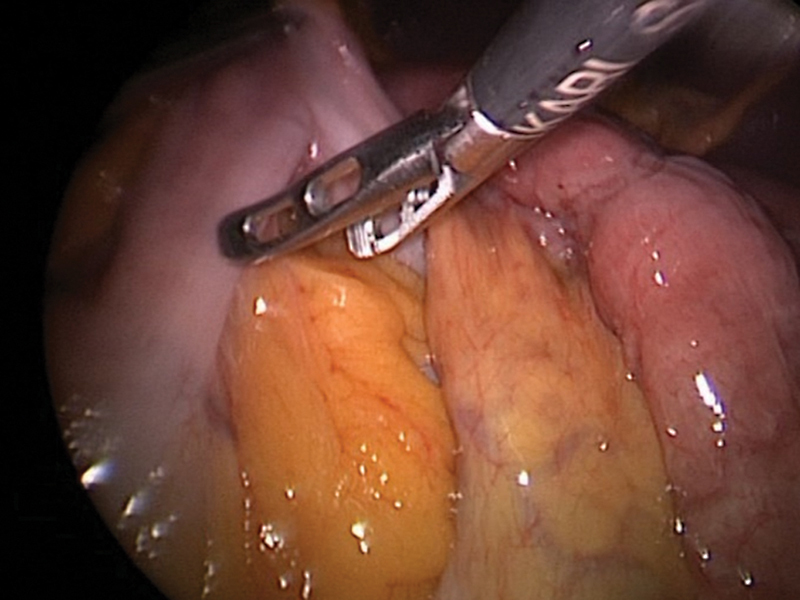
Intraoperative picture demonstrating reduction of internal hernial contents (case 1).

**Fig. 3:**
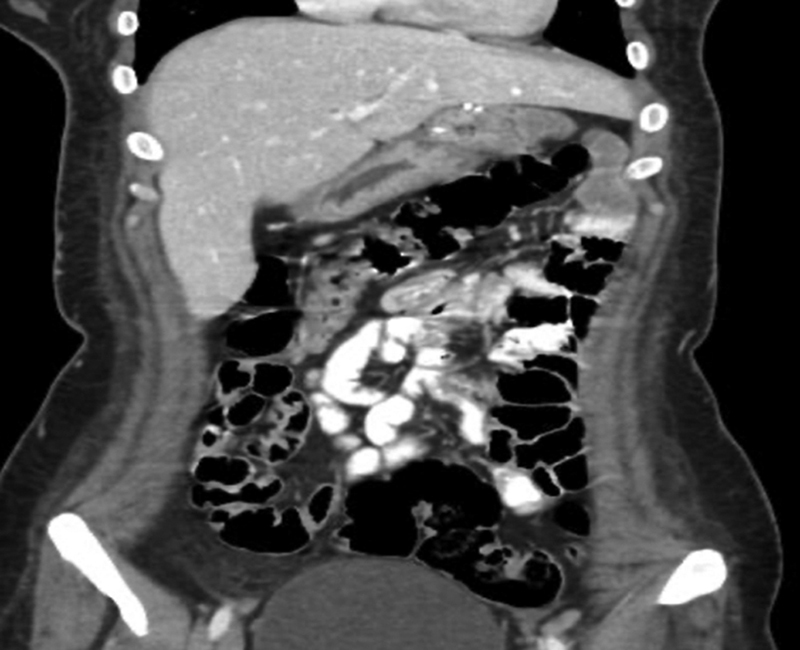
Computed tomography (CT) picture demonstrating swirling of mesentery and distended excluded stomach (case 1).

### Case 2

A 30-year-old female with past surgical history of SAGB + hiatal hernia repair about 1 year ago presented to the clinic with sudden onset, severe colicky abdominal pain. There was no associated nausea, vomiting, abdominal distention, or constipation.

Her vitals were stable and abdomen was soft and lax. There was minimal periumbilical and epigastric tenderness with no guarding or rigidity. Routine biochemistry was within normal range. X-ray abdomen was grossly normal. The only significant finding on CT was a dilated excluded stomach.

Patient underwent diagnostic laparoscopy. Intraoperatively, the biliopancreatic limb was seen entering Petersen's defect leading to twisted mesentery. The biliopancreatic limb was reduced and mesenteric defect was closed. Her postoperative course was uneventful.

### Case 3

A 54-year-old medically free female, with previous SAGB around 8 years ago, presented to the bariatric clinic with persistent refractory reflux.

As part of her symptom evaluation she underwent a gastrografin study which revealed a grade 2 gastroesophageal reflux. An upper GI endoscopy was done which showed postbypass anatomy with wide open lower esophageal sphincter with hiatal hernia with features of bile reflux.

She underwent a diagnostic laparoscopy and the intraoperative findings revealed a grossly distended excluded stomach. Part of small bowel was seen twisted with dilated proximal alimentary limb which was seen entering the Petersen's defect. Laparoscopic reduction with closure of IH defects with conversion of mini-gastric bypass to RYGB was done.

**Fig. 4:**
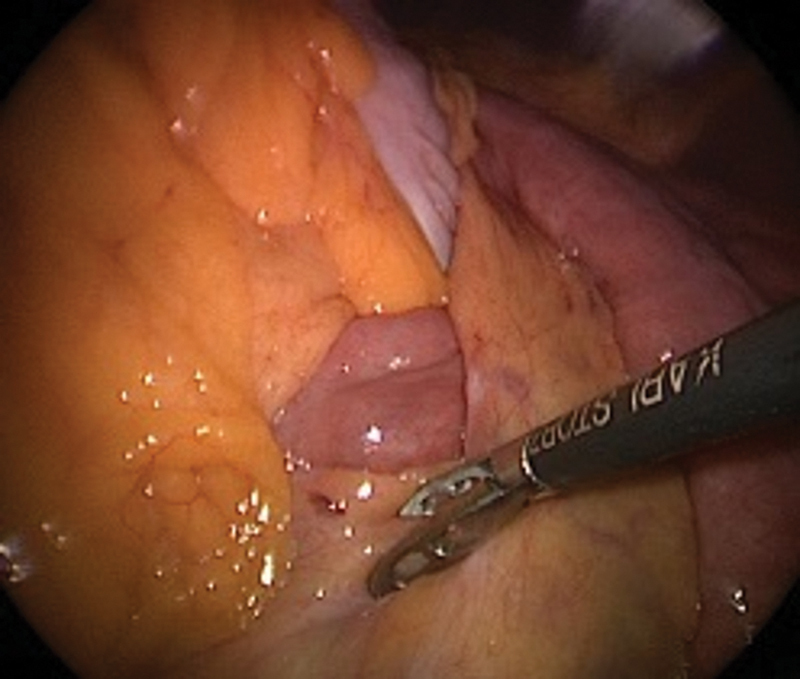
Internal hernia with small bowel as content through the Petersen's defect (case 3).

Intraoperative pictures and CT images shown in Figs. 4 5 6.

**Fig. 5:**
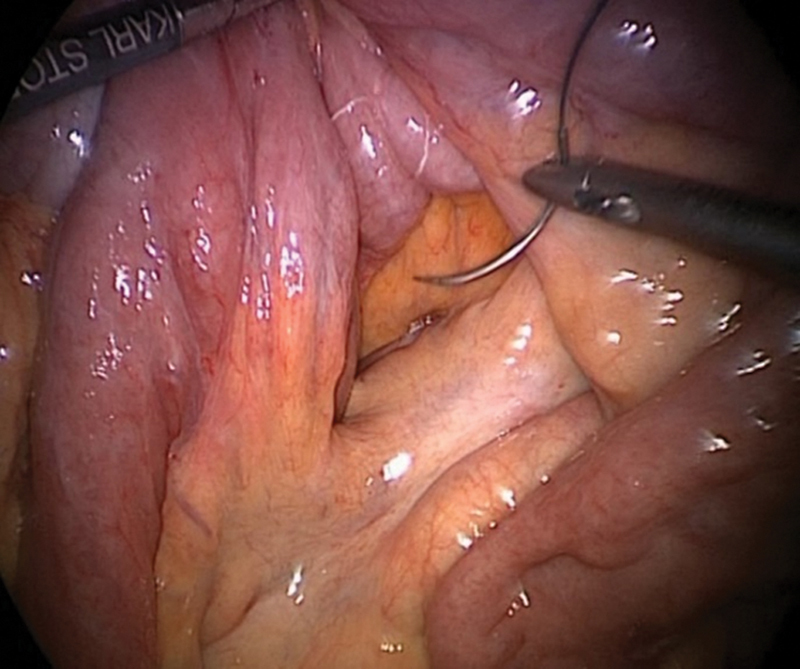
After reduction of contents (case 3).

**Fig. 6:**
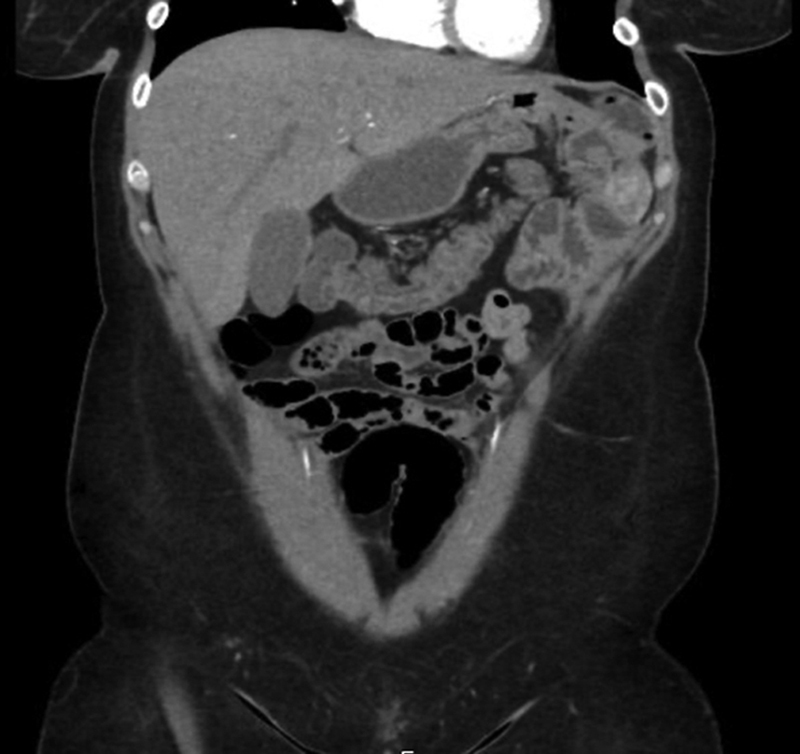
Computed tomography (CT) demonstrating distended excluded stomach (case 3).

## Case 4

A 30-year-old female post-SAGB done 1 year ago, presented to the emergency room with complaints of sudden onset colicky pain abdomen associated with nausea and constipation. There was no history of reflux, vomiting, or abdominal distention.

Her vitals were stable and abdomen soft without any localizing peritoneal signs. Routine biochemical evaluation was unremarkable. CT abdomen revealed a dilated excluded stomach.

Patient underwent diagnostic laparoscopy. Intraoperatively, biliopancreatic limb was seen entering through the Petersen's defect. The bowel was reduced and mesenteric defect was closed. Postoperative period was uneventful.

## Discussion

IH as an entity was defined by Blachar and Federle as protrusion of any viscus (most likely small bowel) through any opening in the mesenteric or peritoneal surfaces, resulting in encapsulation inside another compartment. 2. The IH rate after RYBG is notoriously high and may even reach up to 16%. 3 IH post-SAGB is comparatively rare and is very less frequently reported (0.1–0.4%). 4 5 Schneider et al from their cohort of 934 patients reported that patients who lost more than 90% of their excess weight had very high (nearly twice) risk of developing IHs. 6

Usually, there are three potential spaces through which an IH can form after RYGB. The transmesocolic window through which the Roux limb is taken in a retrocolic approach, the Petersen's defect between the mesentery of the Roux limb and the transverse mesocolon, and the rare jejunojejunal mesenteric defect where the mesenteries of the biliopancreatic limb and the Roux limb meet at the level of jejunojejunostomy. Closure of all peritoneal defects has shown to decrease the incidence of IHs. 7 8 Antecolic position of the Roux limb has been shown to decrease the incidence of IHs as it theoretically eliminates one potential space (transmesocolic) for hernia formation. Koppman et al reported a significantly higher rate of IH after retrocolic versus antecolic positioning of the Roux limb (2.4% vs. 0.3%), respectively ( *p* 0.001) in their case series of 9,500 patients. 9 Similar results have been validated by other studies also. 10 Counterclockwise rotation of the Roux limb to allow the jejunojejunostomy to lie normally on the left side of the mesenteric axis, minimal division of the mesentery, creation of a wide jejunojejunal anastomosis, and placing the omentum on free edges of the Roux limb have all been proposed to decrease the incidence of IHs.

Symptoms of IH post-SAGB are quite nonspecific and depend on the time and extent of herniation. The symptoms can vary from benign intermittent colicky pain to severe intra-abdominal pain presenting as a surgical emergency. Patients with intermittent herniation may report recurrent abdominal cramps with a possible postprandial exacerbation. Acute onset of severe abdominal pain can be indicative of incarceration with threatening small bowl obstruction or infarction. It can be potentially life-threatening if not diagnosed and managed early. IHs can occur any time after surgery. In patients with rapid excessive weight loss (EWS) the highest incidence has been found to be during the initial few years. 2 6

Abdominal examination and routine biochemical investigations are notoriously unreliable in the evaluation of those patients. In their retrospective analysis of 914 patients, Obeid et al noted an IH rate of 5% (45/914). Symptoms attributed to IHs varied from vague postprandial abdominal pain in majority of the patients (53.4%), followed by vague abdominal pain with nausea ± vomiting in 16%; one case (2.2%) presented with acute abdominal pain and peritonitis. 11

Imaging studies form the cornerstone for diagnosis of IH. Their sensitivity is higher especially when done during the presence of symptoms since many IHs can have intermittent symptoms as they may reduce and recur spontaneously. CT is becoming the preferred imaging tool for the evaluation of symptoms suggestive of IH. “Swirly appearance” of the fat and vessels around the mesenteric root, “the hurricane eye” a tubular or round shape of distal mesenteric fat surrounded closely by small bowel loops, “mushroom shape” of the herniated mesenteric root, clustered small bowel loops, and location of small bowel loops (other than the duodenum) behind the superior mesenteric artery are some of the CT-based signs documented in literature that are suspicious for the findings of an IH. Swirling of the mesentery is the single best predictive sign and has been studied extensively. The degree of swirl has been postulated to correlate positively with operative findings with patients having a swirl of 270 degrees having significant intraoperative findings. 12 13 The “swirling,” “mushroom,” “hurricane,” etc. signs suggesting mesenteric twist have to be interpreted with caution as some element of mesenteric rotation is normal after the anatomic alteration associated with SAGB. It is our belief and observation that CT overestimates and overemphasizes the significance of this mesenteric rotation for diagnosis of IH. We recommend cautious interpretation of those signs in close consultation with an experienced radiologist. In our experience, we have noticed that distention of the gastric remnant is a strong surrogate indicator of any downstream partial or complete obstruction and should always be viewed strongly with suspicion. All four of our IH cases had significant dilatation of their gastric remnants.

The first case of an IH after SAGB was reported by Facchiano et al. 14 Petrucciani et al reported one of the largest case series of IH after one-anastomosis gastric bypass with an overwhelming 96/3368 (2.8%) patients developing an IH in their series. They demonstrated the effectiveness of an early surgical referral and timely intervention to prevent any tissue loss. 15 The practice of routine closure of the Petersen's defect as the theoretical elimination of a potential space for formation of IH has proven beneficial after RYGB but has not yet been validated after SAGB. Theoretically, IH after SAGB can be of following three types:

1. IH with biliopancreatic limb herniating through the mesenteric defect
2. IH with alimentary limb as the herniating content
3. IH with both biliopancreatic and alimentary limbs as the content

The management of IH remains primarily surgical. The urgency of surgical intervention is dictated by clinical condition of the patient. The biggest clinical dilemma arises in patients with intermittent symptoms. However, those intermittent symptoms can be recurrent and can even evolve into full blown intestinal obstruction with devastating implications. Hence, surgical intervention in the form of a diagnostic laparoscopy should be offered to patients with intermittent symptoms. Gandhi et al have given those intermittent symptoms the eponym of “Herald symptoms” and found that even with their intermittent nature they have a high degree of operative corelation. 16

Majority of the surgical interventions for IH can be accomplished laparoscopically. Running the whole length of small bowel from fixed points (usually from the ileocecal junction) is done. Reduction of all contents and closure of defects with continuous running or purse-string manner by a nonabsorbable suture is done. Resection and anastomosis is required when bowel viability is questionable. None of the patients in our series required any bowel resection.

The mean follow-up of our operated patients was approximately 16 months. All patients demonstrated relief of their symptoms and are under routine follow-up protocol.

## Conclusion

IH after SAGB have a low incidence rate. The presentation can be acute or delayed. Laparoscopic exploration should be offered to patients with negative imaging but having persistent or recurrent symptoms suggestive of an IH. The experience of the bariatric center, strict follow-up protocols, patient education about possible red flag signs, establishment of a bariatric hotline, and early referral are paramount. Time is tissue and delayed diagnosis can lead to fatal outcomes.
